# Comparing the Electrophysiology and Morphology of Human and Mouse Layer 2/3 Pyramidal Neurons With Bayesian Networks

**DOI:** 10.3389/fninf.2021.580873

**Published:** 2021-02-18

**Authors:** Bojan Mihaljević, Pedro Larrañaga, Concha Bielza

**Affiliations:** Computational Intelligence Group, Departamento de Inteligencia Artificial, Universidad Politécnica de Madrid, Boadilla del Monte, Spain

**Keywords:** partial correlation, inter-species, multivariate, basal, dendrites, allen cell types

## Abstract

Pyramidal neurons are the most common neurons in the cerebral cortex. Understanding how they differ between species is a key challenge in neuroscience. We compared human temporal cortex and mouse visual cortex pyramidal neurons from the Allen Cell Types Database in terms of their electrophysiology and dendritic morphology. We found that, among other differences, human pyramidal neurons had a higher action potential threshold voltage, a lower input resistance, and larger dendritic arbors. We learned Gaussian Bayesian networks from the data in order to identify correlations and conditional independencies between the variables and compare them between the species. We found strong correlations between electrophysiological and morphological variables in both species. In human cells, electrophysiological variables were correlated even with morphological variables that are not directly related to dendritic arbor size or diameter, such as mean bifurcation angle and mean branch tortuosity. Cortical depth was correlated with both electrophysiological and morphological variables in both species, and its effect on electrophysiology could not be explained in terms of the morphological variables. For some variables, the effect of cortical depth was opposite in the two species. Overall, the correlations among the variables differed strikingly between human and mouse neurons. Besides identifying correlations and conditional independencies, the learned Bayesian networks might be useful for probabilistic reasoning regarding the morphology and electrophysiology of pyramidal neurons.

## 1. Introduction

A key challenge in neuroscience is to understand how pyramidal neurons differ across species and cortical regions (Elston et al., 2001; Jacobs and Scheibel, 2002; Benavides-Piccione et al., 2006; Bianchi et al., 2013; Mohan et al., 2015; Gilman et al., 2017; Luebke, 2017). They are often compared in terms of their dendritic morphology, since it directly influences neuronal computation (Häusser et al., 2000; Segev and London, 2000; Spruston, 2008). Compared to rodents, human pyramidal neurons' dendrites are larger (Mohan et al., 2015; Benavides-Piccione et al., 2020; Mihaljević et al., 2020a) and have more synaptic connections per cell (DeFelipe et al., 2002; DeFelipe, 2011), while layers 2 and 3 are easily distinguished in the human and, combined, are thicker than the mouse layer 2/3 (Elston et al., 2001; DeFelipe et al., 2002). Dendritic morphology also varies across cortical regions within a single species (Benavides-Piccione et al., 2002, 2006; Ballesteros-Yáñez et al., 2010; Amatrudo et al., 2012; Mohan et al., 2015; Rojo et al., 2016; Deitcher et al., 2017), although more so in primates than in rodents (Elston, 2003; Gilman et al., 2017; Luebke, 2017). For example, the pyramidal neurons of the monkey visual cortex have smaller basal dendrites than those of its prefrontal cortex, while there is no significant difference in the mouse (Gilman et al., 2017). Deitcher et al. (2017) found that the morphology of human pyramidal neurons also varies with the somatic distance from the pia while that of mouse neurons does not.

In terms of electrophysiology, Gilman et al. (2017) found that mouse visual cortex pyramidal neurons have a lower action potential threshold voltage, shorter action potential rise time, and longer fall time than those of the rhesus monkey, yet found no significant difference in subthreshold features such as time constant and input resistance. Kalmbach et al. (2018), on the other hand, found differences in input resistance and membrane resting potential between human and mouse L2/3 pyramidal neurons, with the degree of difference varying with the somatic distance from the pia. Human cortical neurons have lower membrane capacitance (Eyal et al., 2016) and higher onset rapidity of action potentials than those of adult mouse pyramidal neurons (Testa-Silva et al., 2014). Like morphology, the electrophysiology of pyramidal cells differs across cortical areas (Amatrudo et al., 2012) and age (Zhang, 2004; Elston and Fujita, 2014) and may also vary with somatic distance from the pia within L2/3. In particular, Kalmbach et al. (2018) found such an effect on subthreshold features, including membrane potential and input resistance, in both human and mouse neurons, while Deitcher et al. (2017) found no effect of cortical depth on electrophysiology in human cells (they did not consider the electrophysiology of mouse neurons).

It is well-established that dendritic geometry strongly affects the action potential firing pattern of neurons. For example, given an identical distribution of ion channels over different cortical neuron types, smaller cells tend to spike, whereas larger ones tend to burst (Mainen and Sejnowski, 1996), while computational models suggest that such spiking versus bursting behavior depends on the ratio of somatic surface to dendritic surface (Mason and Larkman, 1990). Also, action potential is accelerated in neurons with larger dendritic surface area (Eyal et al., 2014), which is a likely explanation for the differences in spike onset between human and mouse, given that human dendrites are larger. Computational modeling by Amatrudo et al. (2012) showed that morphological differences between primary visual and prefrontal cortex cells can largely account for differences in passive properties but not in action potential firing nor the synaptic response, thus suggesting differences in active channel conductances. Indeed, the RNA for HCN1, a major pore-forming subunit of h-channels, is ubiquitous in human but not in mouse layers L2/3 (Zeng et al., 2012), and Kalmbach et al. (2018) found that h-channels contribute more prominently to the physiological properties of human pyramidal neurons than to those of the mouse. The differences in h-channel expression, however, could not explain the strong dependence of these electrophysiological properties on cortical depth (Kalmbach et al., 2018), suggesting that one would need to account other factors, including morphology, in order to explain some of the observed cortical depth dependence and inter-species differences.

There have, nonetheless, been relatively little quantitative analyses of how the different electrophysiological and morphological variables correlate with each other and how do these correlations vary between species. An exception is Gilman et al. (2017), who found that larger neurons had a lower input resistance. These analyses were limited to estimating the linear correlation between pairs of variables. This ignores the effect of covariates as well as the conditional (in)dependencies among variables. In other words, relationships often involve more than two variables and thus require a multivariate model; for example, dendritic diameter may be independent of spiking behavior, but when modeled as an exponential function of the distance from the soma, its decay rate is significantly lower for spiker neurons than for bursters and plateauers (Washington et al., 2000). Pairwise analyses can thus be complemented by using multivariate graphical models (Whittaker, 2009) and by quantifying conditional (in)dependencies with partial correlation coefficients. One type of graphical models that is useful for modeling conditional independencies are Bayesian networks (Pearl, 1988; Koller and Friedman, 2009). These models, based on directed acyclic graphs, let us visualize the probabilistic relationships between the variables and are thus useful for exploratory analyses (Bhushan et al., 2019). Their applications in neuroscience (Bielza and Larrañaga, 2014; Bielza and Larrañaga, 2020) include interneuron classification (Mihaljević et al., 2014, 2015; Mihaljević et al., 2019) and the generation of synthetic dendritic branches (López-Cruz et al., 2011).

In this paper, we compare layer 2/3 human temporal cortex and mouse visual cortex pyramidal neurons from the Allen Cell Types Database (http://celltypes.brain-map.org/) in terms of their electrophysiology and dendritic morphology, while assessing the effect of cortical depth on their features. The Allen Cell Type Database cells are unique in that they have been quantified in terms of electrophysiology and morphology, with a standardized procedure for both species. We learn from data Gaussian Bayesian networks in order to identify correlations and conditional independencies between the variables. We learn these networks from three different subsets of our data: (a) from electrophysiological variables alone; (b) from morphological variables alone; and (c) from electrophysiological and morphological variables combined. For each data subset, we learn a Bayesian network per species, which yields a total of six networks; for subset (c), we also show correlation networks (see section 2.6).

The rest of this paper is structured as follows. Section 2 describes the data set, the variables, and analysis methodology. Section 3 provides the results. We discuss our findings in section 4.

## 2. Materials and Methods

### 2.1. Data

We used adult human and adult mouse neurons from the Allen Cell Type Database. Human cells were acquired from donated *ex vivo* brain tissue. We used all excitatory (spiny) cells from layers 2 and 3 of the temporal (human) and visual (mouse) cortex that had a reconstructed morphology. Our sample consisted of 42 human cells from the temporal cortex and 21 mouse cells from the visual cortex.

### 2.2. Electrophysiological Variables

The Allen Cell Type Database provides pre-computed electrophysiological features. These features were derived from high temporal resolution data on membrane potential measurements (in current-clamp mode) obtained with a standardized patch clamp protocol. We used 11 electrophysiological features provided by Allen Cell Type Database, covering subthreshold and suprathreshold features of the cells, including those relative to action potentials. Below we list these features along with brief descriptions (see also Table 1 for their mean values), while we refer the reader to the technical white paper by the Allen Cell Type Database for details (http://help.brain-map.org/download/attachments/8323525/CellTypes_Ephys_Overview.pdf).

**Table 1:** Per-species mean ± standard deviation for each electrophysiological variable, along with the *p*-value of the *t*-test.

**Variable**	**Human**	**Mouse**	***p*-value**
rest (mV)	−71.77 ± 3.75	−77.69 ± 4.06	<0.001
resistance (M Ω)	83.96 ± 54.31	151.61 ± 68.86	<0.001
tau (ms)	27.43 ± 9.01	20.02 ± 9.87	0.006
threshold (pA)	203.33 ± 100.21	186.67 ± 115.94	0.578
peak (mV)	46.31 ± 4.77	37.30 ± 8.68	<0.001
amplitude (mV)	99.36 ± 7.35	91.07 ± 9.07	0.001
up down ratio	3.65 ± 0.81	3.70 ± 0.83	0.822
rise time (μ*s*)	0.56 ± 0.06	0.49 ± 0.06	<0.001
fall time (ms)	0.08 ± 0.17	0.14 ± 0.28	0.355
latency (s)	0.08 ± 0.03	0.06 ± 0.02	0.001
f-i curve (spikes/(s × pA))	0.08 ± 0.05	0.14 ± 0.08	0.004

*<0.001 means that the p-value is below 0.001*.

Subthreshold features were computed as follows: resting potential (rest): average pre-stimulus membrane potential across all the long square responses; input resistance (resistance): the slope of a linear fit of minimum membrane potentials during the responses onto their respective stimulus amplitudes for long square sweeps with negative current amplitudes that did not exceed 100 pA; time constant (tau): exponential curve fit between 10% of the maximum voltage deflection (in the hyperpolarizing direction) and the minimum membrane potential during the response, and the time constants of these fits were averaged across steps to estimate the membrane of the cell.

All action potential waveforms were evoked by a long square (1 s) current step stimulus. The waveforms of the first action potentials were collected from each cell and aligned on the time of their thresholds. Action potential features were computed as follows: threshold (threshold): the level of injected current at threshold; peak (peak): maximum value of the membrane potential during the action potential; amplitude (amplitude): difference between the action potential trough and the action potential peak, where the trough is the minimum value of the membrane potential between the peak and the next action potential; upstroke/downstroke ratio (up down ratio): the ratio between the absolute values of the action potential peak upstroke and the action potential peak downstroke, where the upstroke is the maximum value of dV/dt between the threshold and the peak, and peak downstroke is the minimum value of dV/dt between the peak and the trough; rise time (rise time): time from threshold to the peak; fall time (fall time): time from peak to the trough.

Additional suprathreshold features were computed as follows: latency (latency): time between the start of the stimulus until the first spike; “f-i curve” (f-i curve): slope of a straight line fit to the suprathreshold part of the curve of frequency response of the cell versus stimulus intensity for long square responses.

### 2.3. Morphological Variables

The Allen Cell Type Database provides 3D neuron morphology reconstructions. These were obtained by filling the cells with biocytin and serially imaged to visualize their morphologies. Detailed description of the reconstruction protocol is provided in the Allen Cell Type Database morphology overview technical whitepaper (http://help.brain-map.org/download/attachments/8323525/CellTypes_Morph_Overview.pdf).

We computed nine features of both basal and apical dendrites. Of these features, four are arbor-level features, whereas five are branch- or bifurcation-level features. We computed the features with the open-source NeuroSTR library (https://computationalintelligencegroup.github.io/neurostr/). Below we list these features along with brief descriptions (see also Table 2 for their mean values). The variable names provided in parenthesis correspond to basal dendrite variables; the corresponding variable of the apical dendrite is denoted with an a prefix: for example, a.distance instead of distance.

**Table 2:** Per-species mean ± standard deviation for each morphological variable, along with the *p*-value of the *t*-test.

**Variable**	**Human**	**Mouse**	***p*-value**
distance (μ*m*)	133.00 ± 63.00	63.00 ± 5.00	<0.001
length (μ*m*)	81.00 ± 15.00	44.00 ± 6.00	<0.001
tortuosity	1.15 ± 0.05	1.14 ± 0.05	0.518
angle (rad)	1.01 ± 0.14	1.13 ± 0.18	0.012
diameter (μ*m*)	0.33 ± 0.12	0.27 ± 0.06	0.009
height (μ*m*)	403.00 ± 262.00	192.00 ± 22.00	<0.001
width (μ*m*)	423.00 ± 147.00	227.00 ± 48.00	<0.001
depth (μ*m*)	114.00 ± 26.00	62.00 ± 17.00	<0.001
total_length (μ*m*)	5008.00 ± 3108.00	1867.00 ± 540.00	<0.001
a.distance (μ*m*)	285.00 ± 64.00	147.00 ± 26.00	<0.001
a.length (μ*m*)	99.00 ± 15.00	55.00 ± 12.00	<0.001
a.tortuosity	1.13 ± 0.05	1.14 ± 0.04	0.653
a.angle (rad)	0.89 ± 0.12	1.20 ± 0.17	<0.001
a.diameter (μ*m*)	0.36 ± 0.12	0.28 ± 0.06	0.003
a.height (μ*m*)	596.00 ± 213.00	262.00 ± 81.00	<0.001
a.width (μ*m*)	525.00 ± 146.00	274.00 ± 85.00	<0.001
a.depth (μ*m*)	122.00 ± 27.00	85.00 ± 31.00	<0.001
a.total_length (μ*m*)	5352.00 ± 1838.00	1480.00 ± 702.00	<0.001

*<0.001 means that the p-value is below 0.001*.

The branch-level features were averaged across all bifurcations or branches of an arbor and were computed as follows: average branch length (length): sum of the lengths of all compartments of a branch, averaged over all bifurcation points; average path distance (distance): sum of the lengths of all compartments' length starting from the dendrites' insertion point into the soma up the bifurcation point, averaged over all bifurcation points; average branch tortuosity (tortuosity): ratio of branch length and the length of the straight line between the beginning and the end of a branch, averaged over all branches; average remote bifurcation angle (angle): shortest planar angle between the vectors from the bifurcation to the endings of the daughter branches, averaged across all bifurcations; average branch diameter (diameter).

Arbor-level features were computed as follows: height (height): difference between the maximum and minimum values of Y-coordinates of the dendrites; width (width): difference between the maximum and minimum values of X-coordinates of the dendrites; depth (depth): difference between the maximum and minimum values of Z-coordinates of the dendrites; total length (totallength): sum of branch length of all the branches of the dendrites.

**Figure 1:**
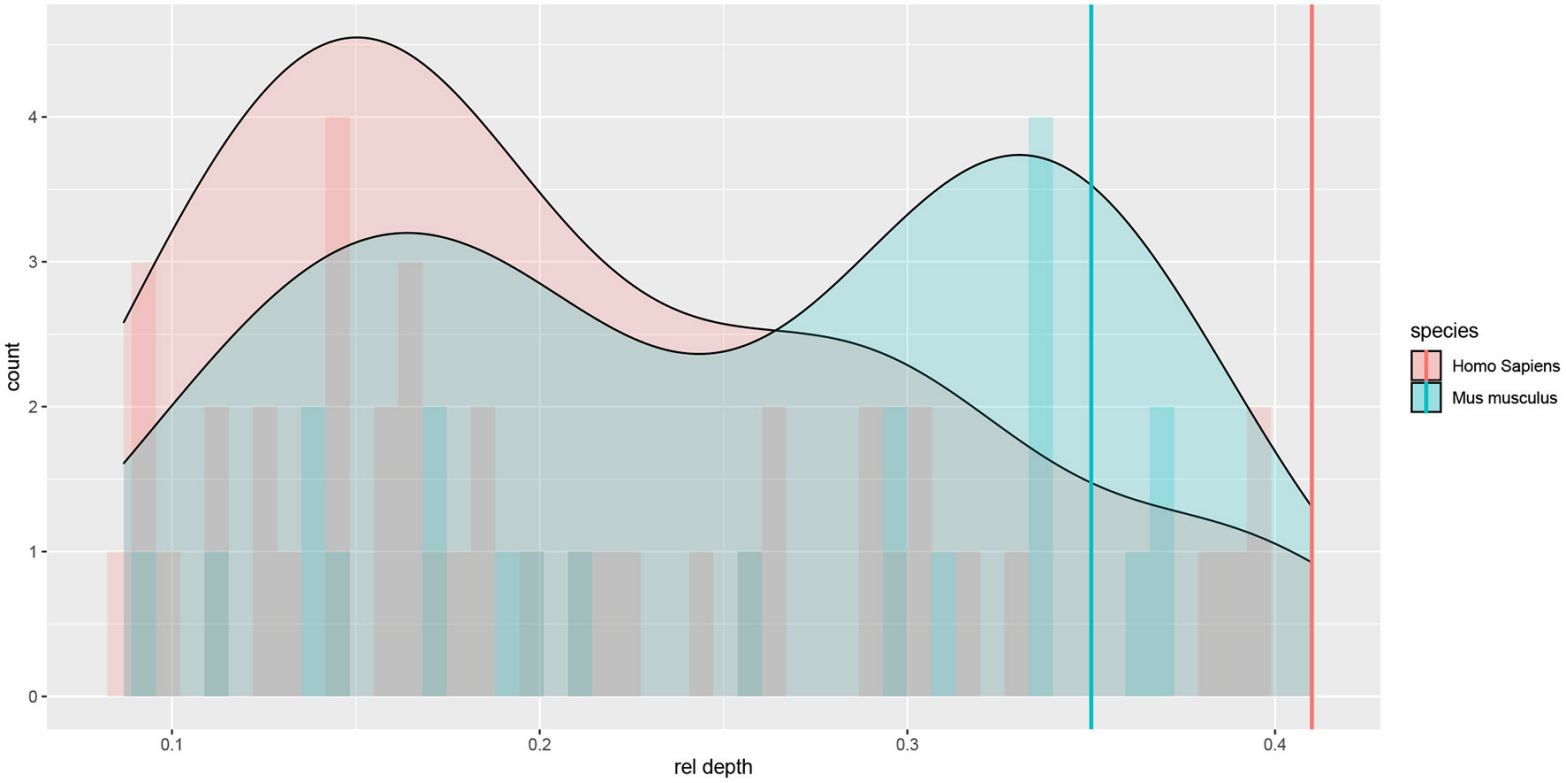
Histograms and density plots of the cells' relative (to pia and white matter) cortical depth. Depths close to 0 denote superficial cells. Vertical lines denote rough estimates of the boundaries of L4 in the two species, given by the cortical depth of the most superficial L4 cell that we observed among Allen Cell Type Database neurons.

The Allen Cell Type Database provided the depth of each cell's soma (rel depth) relative to pia and white matter. There were both superficial and deep cells in both species; although deep cells were better represented in the mouse sample, whereas superficial ones in the human sample (see Figure 1).

### 2.4. Bayesian Networks

A Bayesian network (BN) (Koller and Friedman, 2009) B allows us to compactly encode a joint probability distribution over a vector of *n* random variables **X** by exploiting conditional independencies among triplets of sets of variables in **X** (e.g., *X* is independent of *Y* given *Z*). A BN consists of a directed acyclic graph (DAG) G and a set of parameters **θ** [B=(G,θ)]. The vertices (i.e., nodes) of G correspond to the variables in **X**, while its directed edges (i.e., arcs) encode the conditional independencies among **X**. A joint probability density $f_{G}\left(\text{x}\right)$ encoded by B, where **x** is an assignment to **X**, factorizes as a product of local conditional densities,

$$f_{G}(x)=\prod_{i=1}^{n}f_{G}(x_{i}|pa_{G}\left(x_{i}\right)),$$

where ${\text{p}\text{a}}_{G}\left(x_{i}\right)$ is an assignment to variables ${\text{P}\text{a}}_{G}\left(X_{i}\right)$, the set of parents of *X*_*i*_ in **X** according to G. G induces conditional independence constraints for $f_{G}\left(\cdot \right)$, derivable from the basic constraints that each *X*_*i*_ is independent of its nondescendents in G given ${\text{P}\text{a}}_{G}\left(X_{i}\right)$. For example, for any pair of variables *X, Y* in **X** that are not connected by an arc in G there exists a set of variables **Z** in **X** (disjoint from {*X*} and {*Y*}) such that *X* and *Y* are independent conditionally on **Z** [i.e., $f_{G}\left(X,Y|\text{Z}\right)=f_{G}\left(X|\text{Z}\right)f_{G}\left(Y|\text{Z}\right)$]. Similarly, for any pair of variables *X, Y* in **X** that are connected by an arc in G there is no set **Z** such that *X* and *Y* are independent conditionally on **Z**. These constraints extend to nodes not connected by an arc in G and the structure G thus lets us identify conditional independence relationships among any triplet of sets of variables *X*, *Y*, and **Z** in **X**. For example, in the DAG *X* → *Y* → *Z* we only have one independence: *X* is independent of *Z* conditional on *Y*; *X* and *Y*, *X* and *Z*, and *Y* and *Z* are not marginally independent. The Markov blanket of *X*_*i*_ is the set of variables **MB**(*X*_*i*_) such that *X*_*i*_ is independent of **X** \**MB**(*X*_*i*_) conditional on **MB**(*X*_*i*_). The Markov blanket of *X*_*i*_ is easily determined from G as it corresponds to the parents, the children, and the spouses (other parents of the children of *X*_*i*_) of *X*_*i*_ in G.

The parameters **θ** specify the local conditional densities $f_{G}(x_{i}|pa_{G}(x_{i}))$ for each variable *X*_*i*_. When **X** contains only continuous variables, as in our case, a common approach is to let $f_{G}\left(\text{x}\right)$ be a multivariate normal density. The local conditional density for *X*_*i*_ is $f_{G}(x_{i}|pa(x_{i}))=N(x_{i};\beta_{i0}+\beta_{i}^{T}pa_{G}(x_{i}),\sigma_{i}^{2})$. There is thus a different vector of coefficients $\left(\beta_{i0},\beta_{i}^{T},\sigma_{i}^{2}\right)$ for each *X*_*i*_.

Two or more DAGs can encode the same set of conditional independencies. A set of such equivalent DAGs can be uniquely represented with a completed partially directed graph (CPDAG). An edge between *X* and *Y* is directed in the corresponding CPDAG only if it is identically oriented in every equivalent DAG; it is undirected otherwise.

### 2.5. Learning Bayesian Networks From Data

Learning a Bayesian network B from a data set $D=\left\{{\text{x}}^{1},\ldots ,{\text{x}}^{N}\right\}$ of *N* observations of **X** involves two steps: (a) learning the DAG G and (b) learning **θ**, the parameters of the local conditional distributions. There are two main approaches to learning G from D (Koller and Friedman, 2009): (a) by testing for conditional independence among triplets of sets of variables (the *constraint-based* approach); and (b) by searching the space of DAGs in order to optimize a score such as penalized likelihood (the *score-based* approach). While seemingly very different, conditional independence tests and network scores are related statistical criteria (Scutari et al., 2019). For example, when considering whether to include the arc *Y* → *X* into a graph G, the likelihood-ratio test of conditional independence of *X* and *Y* given ${\text{P}\text{a}}_{G}\left(X\right)$ and the Bayesian information criterion (Schwarz, 1978) (BIC) score are both functions of $\log\frac{P\left(X|{\text{P}\text{a}}_{G}\left(X\right),Y\right)}{P\left(X|{\text{P}\text{a}}_{G}\left(X\right)\right)}$. They differ in computing the threshold for determining independence: the former relies on the distribution of the statistic under the null model (i.e., conditional independence), whereas the latter is based on an approximation to the Bayes factor between the null and alternative models. Besides using different criteria, the constraint-based and score-based approaches also differ in model search, that is, in terms of the sets *X*, *Y*, and **Z** that they choose to test conditional independence. The score-based approaches tend to be more robust (Koller and Friedman, 2009), as they may reconsider previous steps in the search by removing or reversing previously added arcs. We thus followed a score-based approach in this paper.

#### 2.5.1. Confidence in Network Structure

A difficulty for inferring the network structure is that the number of data instances is relatively small compared to the number of variables, especially for the mouse data set. This might result in many different high-scoring structures and thus reduces the confidence in a particular learned structure. In order to paliate this, we use the bootstrap-based (Efron, 1979) approach by Friedman et al. (1999) to filter out arcs that are likely to be spurious. In particular, we begin by taking *B* samples from the empirical distribution and apply our learning algorithm on each sample to produce *B* Bayesian networks. The confidence in the arc *X* → *Y*, *p*(*X* → *Y*), is then estimated as the fraction of times that *X* → *Y* appears in the *B* networks. We then consider that all arcs *X* → *Y* with *p*(*X* → *Y*) + *p*(*X*←*Y*) ≤ *t*, where *t* is some threshold, are spurious and thus blacklist them when learning the definitive network structure.

A reasonable threshold *t* might be 0.5, so that we discard all arcs which we find more likely to be spurious than not. By experimenting with synthetic data, we found, indeed, that the confidence estimates of nonspurious arcs were never below 0.48. On the other hand, the confidence estimates for spurious arcs tended to be inflated, with a maximum of 0.86 and the third quartile around 0.5. We thus used *t* = 0.7 as it provided none or few false positives in our experiments while yielding reasonably few false negatives.

We found that the above procedure filtered out most of the possible arcs in each of the six networks, leaving few candidate arcs for the definitive structure learning. Note that we considered both *X* → *Y* and *X* ← *Y* in order to compute the confidence in a direct link between *X* and *Y*. This is because we found that arc directions were rarely established with confidence and we thus filter out arcs that have insufficient confidence in the directions combined.

### 2.6. Marginal and Partial Correlation Coefficients

For Gaussian variables, the partial correlation coefficient ρ_*XY*∣**Z**_ of *X* and *Y* given all other variables **Z** = **X** \ {*X, Y*} equals the correlation between the residuals *R*_*X*_ = *X* − *f*_*X*_(**Z**) and *R*_*Y*_ = *Y* − *f*_*Y*_(**Z**), where *f*_*X*_(**Z**) is a linear regression of *X* onto **Z** and likewise for *f*_*Y*_(**Z**). The ρ_*XY*∣**Z**_ can be computed directly from the inverse of the joint covariance matrix Σ, $\rho_{XY|\text{Z}}=-\frac{\Omega_{XY}}{\sqrt{\Omega_{XX}\Omega_{YY}}}$, where Ω = Σ^−1^. By estimating Ω from data, we estimate pairwise conditional independencies, since ρ_*XY*∣**Z**_ = 0 (and thus Ω_*XY*_ = 0) if and only if *X* and *Y* are independent conditional on **Z**. One way to estimate Ω is by learning a Bayesian network from the data. Namely, for standardized variables **X**, Ω = (*I* − *B*)*S*^−1^(*I* − *B*)^*T*^ (Aragam and Zhou, 2015), where *B* is the matrix containing the network's parameters with each **β**_*i*_ in one column, *B* = [**β**_1_|⋯|**β**_*n*_], and *S* the diagonal matrix containing variances of local conditional distributions $S_{ii}=\sigma_{i}^{2}$. The estimate is then ${\hat{\Omega }}_{B}=\left(I-\hat{B}\right){Ŝ}^{-1}{\left(I-\hat{B}\right)}^{T}$, where $\hat{\cdot }$ denotes the empirical estimate. Note that the Bayesian network provides an estimate of Ω even when the empirical correlation matrix $\hat{\Sigma }$ is not invertible (e.g., when *n* > *N*).

The heavy regularization of Bayesian networks with bootstrap blacklisting shrinks many marginal correlations correlation coefficients ρ in the correlation matrix associated with the Bayesian network, ${\hat{\Sigma }}_{B}={\hat{\Omega }}_{B}^{-1}$ to 0. We thus report correlation coefficients derived from the empirical $\hat{\Sigma }$, rather than those derived from the ${\hat{\Sigma }}_{B}$. Note that marginal correlations are easily seen on a correlation network, an undirected graph that has an arc between *X* and *Y* if the absolute value of their correlation is above some threshold.

### 2.7. Comparing Bayesian Networks

We used the Hellinger distance (Pardo, 2018) in order to quantify the difference between Bayesian network structures of the two species. This is a bounded metric for probability distributions, with a value of 0 for identical distributions and a maximum distance of 1. As such, it also depends on the parameters of the Bayesian network; for example, it can be high for two normal distributions with identical structures with very different means, meaning that we could have a large distance simply due to inter-species differences in the variables' magnitudes (Tables 1, 2). We thus isolated the effect of inter-species differences in the means by re-fitting the parameters of one of the distributions before the comparison. Namely, we re-fit the parameters of the human Bayesian network on the mouse data before comparing it to the mouse Bayesian network; likewise, we re-fit the parameters of the mouse Bayesian network on the human data before comparing it with the original human Bayesian network. This means that we report two Hellinger distance values, one from each (human and mouse) data set. Note that the means of the compared distributions are always the same, as they are estimated from the same data set. The Hellinger distance *H* is a function of the estimated covariance matrices only, $H\left(B_{1},B_{2}\right)={\left(1-\frac{\det{\left({\hat{\Sigma }}_{B_{1}}\right)}^{1/4}\det{\left({\hat{\Sigma }}_{B_{2}}\right)}^{1/4}}{\det{\left(\frac{{\hat{\Sigma }}_{B_{1}}+{\hat{\Sigma }}_{B_{2}}}{2}\right)}^{1/2}}\right)}^{1/2},$ where det denotes a matrix determinant.

### 2.8. Settings

We used *B* = 2, 000 bootstrap samples for estimating arc confidence and blacklisted all arcs with an estimated confidence below 0.7. We then learned network structures by using the tabu algorithm (Glover and Laguna, 2013), implemented in the bnlearn R package (Scutari, 2010; R Core Team, 2015), to optimize the BIC score. The tabu algorithm is a local search that efficiently allows for score-degrading operators by avoiding those that undo the effect of recently applied operators; we used a tabu list of size 30 and allowed for up to 30 iterations without improving network score.

## 3. Results

We first look at electrophysiological (section 3.1) and morphological features (section 3.2) separately, and then at joint Bayesian networks and correlation networks for both electrophysiological and morphological features (section 3.3).

### 3.1. Electrophysiology

All variables except for threshold, up down ratio, and fall time differed significantly between the species (Table 1). Human neurons had lower a resistance, higher time constant (tau), rest potential, peak action potential voltage, amplitude and latency, and a longer action potential rise time.

**Figure 2:**
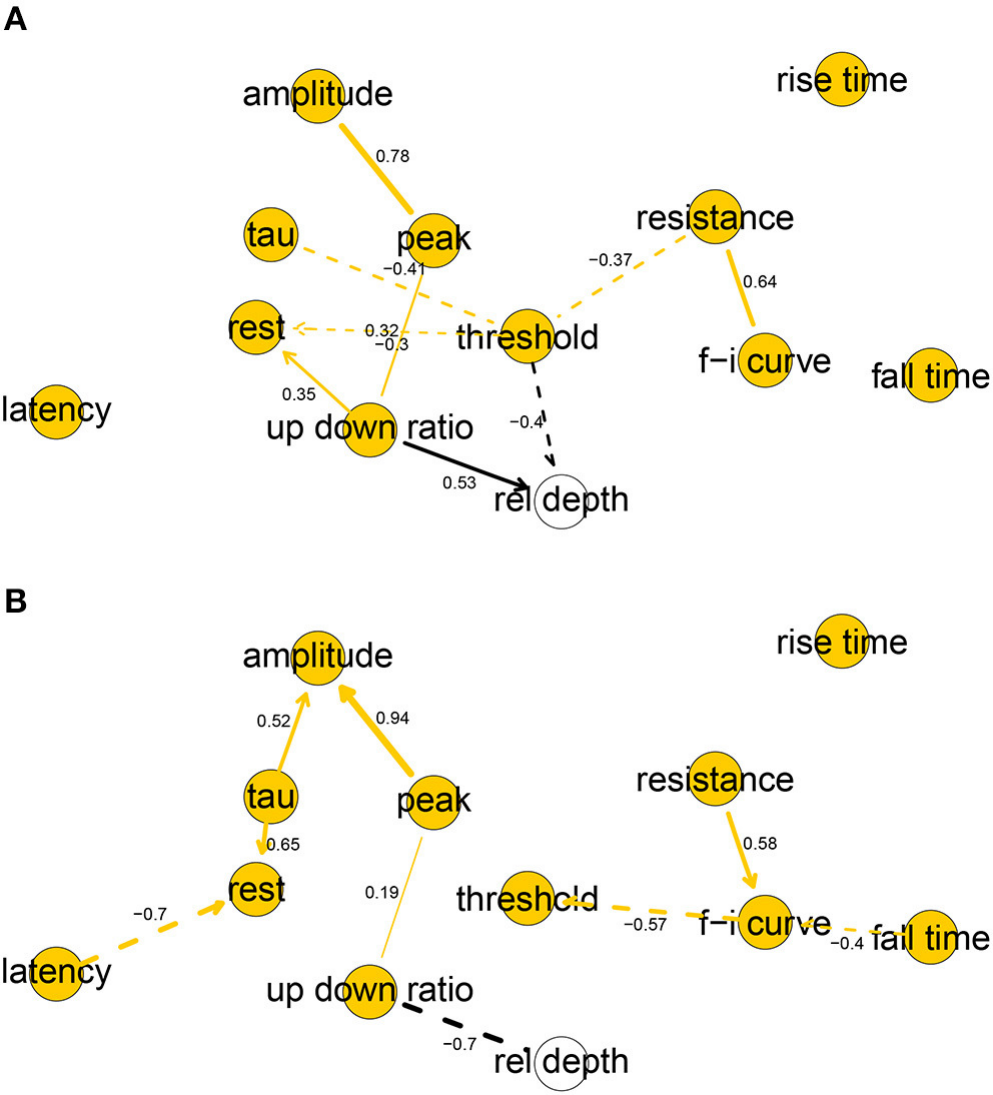
Completed partially directed graphs (CPDAGs) of the Bayesian networks for electrophysiological features of human **(A)** and mouse **(B)** cells. Arc width is proportional to the absolute value of the partial correlation (shown next to the arc) between the nodes. Arcs corresponding to negative partial correlations plotted with dashed lines. Proximity between two nodes is unrelated to the magnitude of partial correlation.

The human and mouse BNs uncovered relevant correlations and independencies among the variables. In the human BN (Figure 2A), the Markov blanket of rel depth consisted of threshold and up down ratio, while it was marginally correlated with all variables except for latency, fall time, and rise time. In particular, rel depth had a strong positive marginal (0.59) and partial correlation with up down ratio (0.53) and a strong negative one with threshold (−0.40). This is contrary to the results of Deitcher et al. (2017) who found that human electrophysiological features such as input resistance and membrane time constant were independent of depth in the human L2/3 pyramidal neurons of the temporal cortex and, on the other hand, is partially consistent with the results of Kalmbach et al. (2018) (see section 4). Variables fall time, rise time, and latency were each uncorrelated with all other variables, f-i curve was independent of all other variables given resistance, as were tau given threshold and amplitude given peak. All other variables had Markov blankets of size two or larger, with the largest being that of threshold with five variables. The strongest partial correlations were those between peak and amplitude (0.78) and resistance and f-i curve (0.64). See Figure 2A for non-zero all partial correlation coefficients.

In the mouse BN (Figure 2B), rel depth was correlated with up down ratio and peak, amplitude, and fall time, while its Markov blanket contained only up down ratio. Contrary to the human BN, its marginal (−0.84) and partial (−0.54) correlation with up down ratio was strongly negative. rise time was uncorrelated with other variables, and resistance and threshold were independent of all other variables given f-i curve. The remaining variables had Markov blankets of size two or larger, with the largest being that of f-i curve with four variables. The strongest partial correlations were those between peak and amplitude (0.94), and latency and rest (−0.70). See Figure 2B for all nonzero partial correlation coefficients.

Overall, the human and mouse BNs were strikingly different, with only two common arcs in their CPDAGs (resistance—f-i curve and peak—up down ratio). No variable had an identical Markov blanket in the two graphs and Hellinger distances on human and mouse data sets, respectively, were 0.44 and 0.61. While the magnitudes of threshold, fall time, and up down ratio did not differ significantly between the species (Table 1), the BNs show that their correlations with other variables did. A rare common feature of the two BNs was the strong positive partial correlation between amplitude and peak.

### 3.2. Morphology

All variables, except for tortuosity, differed significantly between the two species (Table 2). Human dendrites were larger, had longer and thicker branches and, especially in apical dendrites, sharper bifurcation angles. Deitcher et al. (2017), on the contrary, report similar branch diameter in human and mouse neurons. The average human apical arbor was 3.6 times longer than the mouse one, while the average human basal arbor was 2.7 times longer. This is more pronounced than the 3.2-fold and 2.1-fold differences that Mohan et al. (2015) observed for apical and basal dendrites, respectively, of human and mouse temporal cortex pyramidal neurons.

**Figure 3:**
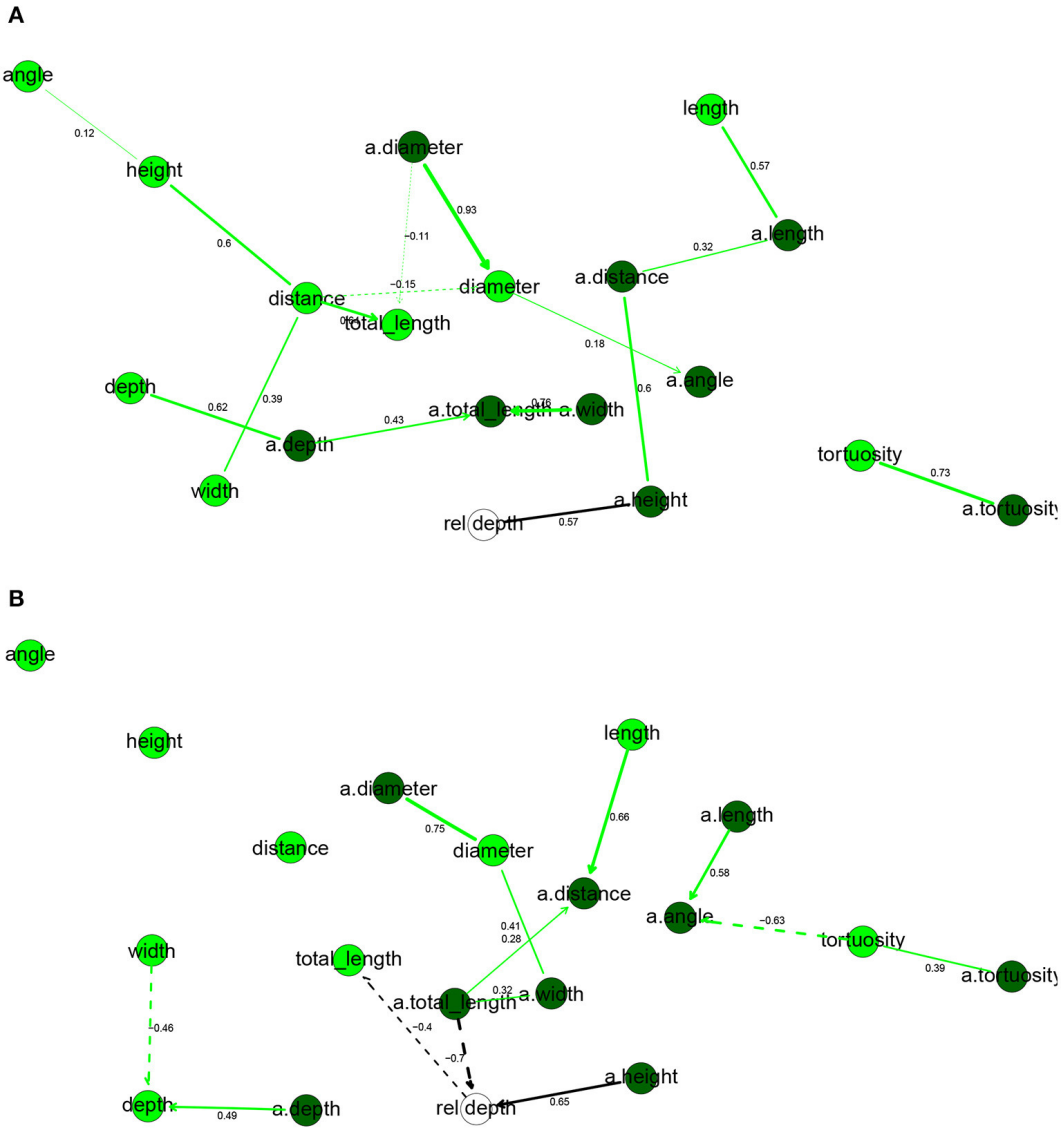
Completed partially directed graphs (CPDAGs) of the Bayesian networks for morphological features of human **(A)** and mouse **(B)** cells. Basal nodes are in green and apical nodes are in dark green. Arc width is proportional to the absolute value of the partial correlation (shown next to the arc) between the nodes. Arcs corresponding to negative partial correlations plotted with dashed lines. Proximity between two nodes is unrelated to the magnitude of partial correlation.

In the human BN (Figure 3A), rel depth had only a.height in its Markov blanket, while it was also correlated with a.distance, a.length, and length but independent of the remaining variables, including a.totallength (marginal correlation coefficient ρ = 0.34) and totallength (ρ = 0.03). Thus, while the height of the apical arbor increased significantly with depth from the pia, total arbor length did not. These results are contrary to those of Deitcher et al. (2017), who found strong correlations between depth from the pia and a number of apical and basal variables, including basal dendrites' total length (ρ = 0.50) and apical arbor width (0.48). We found that most basal dendrites' variables were positively correlated with the corresponding apical variable, with the exceptions being the bifurcation angles and the distance from soma. The diameter was particularly consistent, with ρ = 0.94 between diameter and a.diameter.

In the mouse BN (Figure 3B), the Markov blanket of rel depth contained totallength, a.totallength, and a.height, while it was marginally correlated also with diameter, a.height, a.width, a.distance, and a.diameter. This is contrary to the results that Deitcher et al. (2017) observed on temporal cortex mouse cells, as they found no significant change in morphological features with increasing depth. While a.height increased with rel depth, a.totallength decreased strongly with rel depth, both marginally (ρ = −0.83) and conditionally on all other variables (ρ_*XY*∣**Z**_ = −0.66). We observed the same, yet slightly weaker, effect for basal dendrites (ρ = −0.73 and ρ_*XY*∣**Z**_ = −0.40 with totallength). Thus, deeper mouse cells had smaller apical and basal arbors and, perhaps surprisingly, this was in spite of them having higher apical arbors. As in human cells, basal variables were often correlated with the corresponding apical variables. Unlike in the human, cells with larger basal dendrites tended to have thicker branches (ρ = 0.43) while a.angle had a negative partial correlation with a.diameter.

Overall, the human and mouse BNs were strikingly different, with only one common arc in their CPDAGs (tortuosity—a.tortuosity ). Only a.tortuosity had an identical Markov blanket in the two graphs. The Hellinger distances were larger than for electrophysiological variables, with a value of 0.87 on the human data set and 0.75 on the mouse data set.

### 3.3. Electrophysiology and Morphology

**Figure 4:**
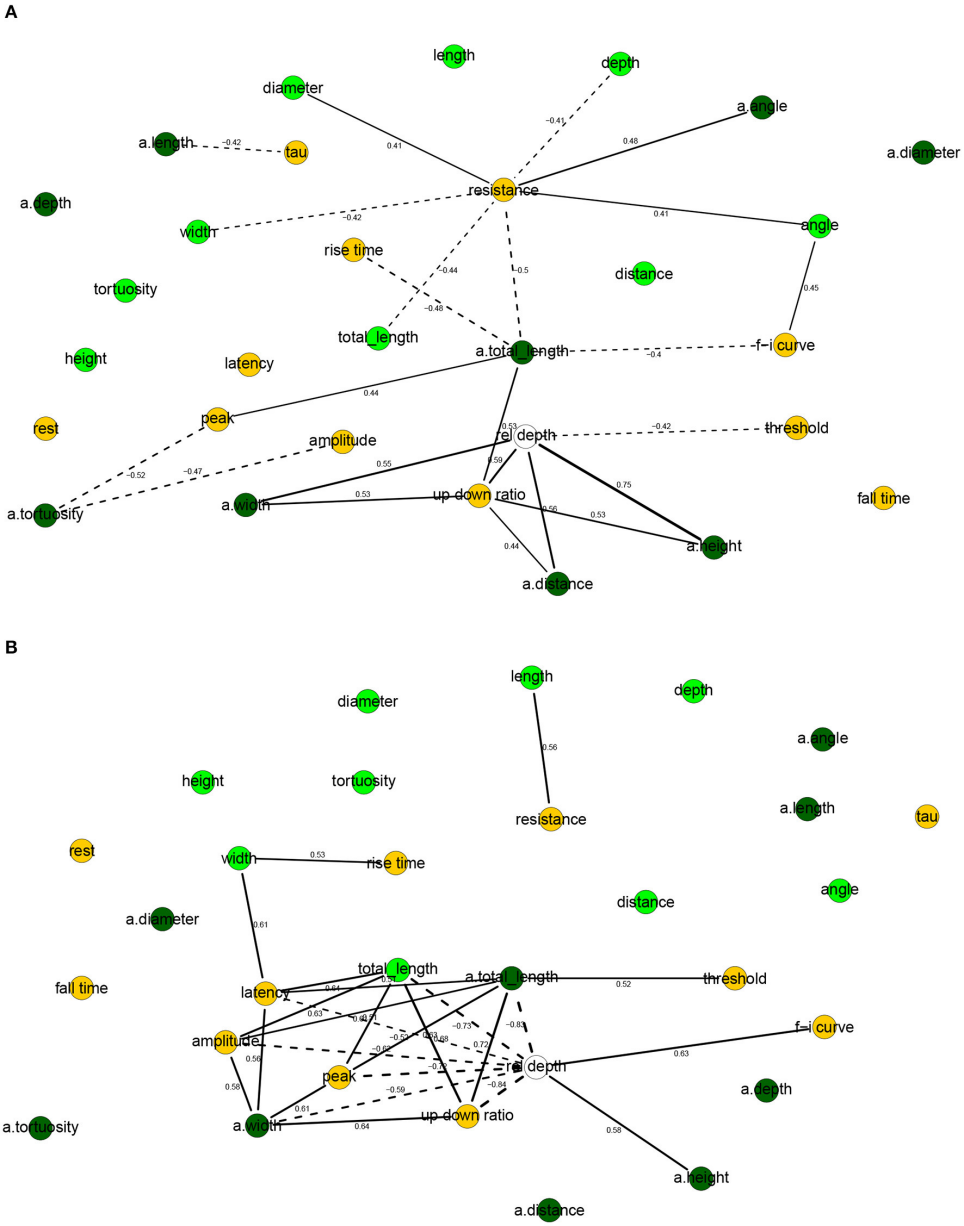
Correlation networks for electrophysiological and morphological features of human **(A)** and mouse **(B)** cells. Showing only arcs between morphological and electrophysiological variables as well arcs to/from rel depth and with an absolute correlation above 0.4 for human cells and 0.5 for mouse cells. These threshold values were well above the 0.05 significance level and thus correspond to strong correlations. Morphological nodes are shown in green, with apical nodes in dark green; electrophysiological nodes in orange.

**Figure 5:**
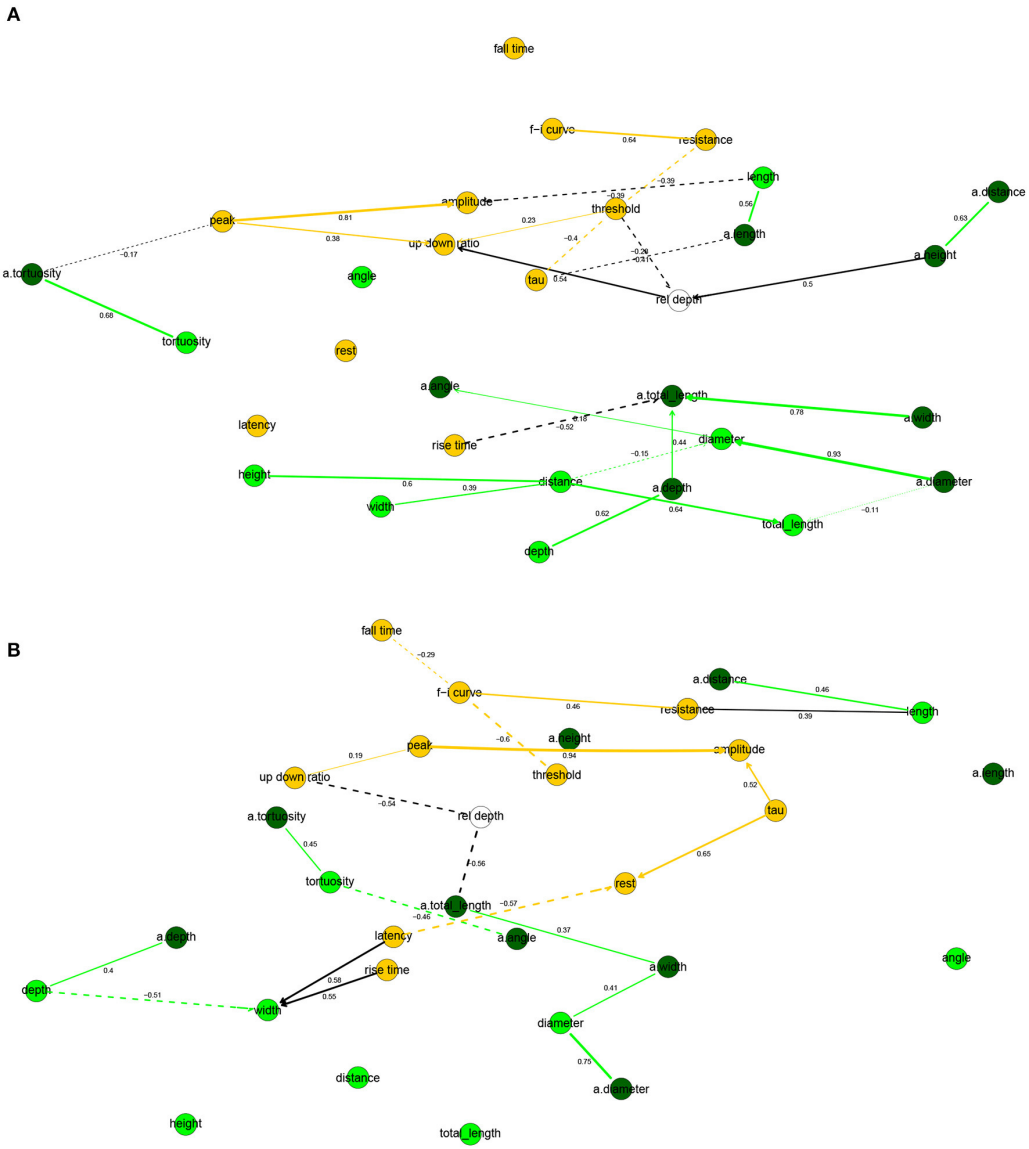
Completed partially directed graphs (CPDAGs) of the Bayesian networks for electrophysiological and morphological features of human **(A)** and mouse **(B)** cells. Morphological nodes and the arcs between them shown in green, with apical nodes in dark green; electrophysiological nodes and the arcs between them in orange. Arc width is proportional to the absolute value of the partial correlation (shown next to the arc) between the nodes. Arcs corresponding to negative partial correlations plotted with dashed lines. Proximity between two nodes is unrelated to the magnitude of partial correlation.

The correlation networks (Figure 4) and the Bayesian networks (Figure 5) show many correlations between electrophysiological and morphological variables.

In human cells, all electrophysiological variables except for latency, fall time, and rest were marginally correlated with at least one morphological variable (Figures 4A, 5A). Besides features related to arbor size, electrophysiological variables were also correlated with branch-level features such as the mean bifurcation angle. While up down ratio was strongly correlated with features of apical arbor size (e.g., ρ = 0.53 with a.totallength), these correlations were explained away by the cortical rel depth and hence up down ratio was independent in the BN, conditional on its Markov blanket, of all morphological variables. Interestingly, peak decreased strongly (ρ = −0.52) with a.tortuosity and this effect persisted after conditioning on the remaining variables (ρ_*XY*∣**Z**_ = −0.17). Input resistance was negatively correlated with basal and apical arbor size (e.g., ρ = −0.50 with a.totallength and ρ = −0.44 with totallength). While it is already known that resistance decreases with dendritic size (Gilman et al., 2017), we found that it decreased additionally (ρ_*XY*∣**Z**_ = −0.30) with basal arbor width after accounting for totallength. As in Figure 2A, rise time was independent of all electrophysiological variables; it was, however, correlated with morphological ones. In particular, rise time decreased with apical a.totallength (ρ = −0.48, ρ_*XY*∣**Z**_ = −0.49) and increased with basal bifurcation angle (ρ = 0.37, ρ_*XY*∣**Z**_ = −0.32). The Markov blanket of rel depth contains up down ratio, threshold, as in Figure 2A, as well as a.height, as in Figure 3A. Since rel depth is not independent of the electrophysiological variables given the morphological ones, Figure 5A shows that the correlation of rel depth with the electrophysiological variables cannot be explained as an indirect effect of the differences in morphology with respect to cortical depth, and instead corresponds to an effect of cortical depth on electrophysiology that is not explained by our morphological variables.

In mouse cells, there were also many marginal correlations between electrophysiological and morphological variables, with 16 arcs between electrophysiological and morphological features in the correlation network (Figure 4B) and 3 in the Bayesian network (Figure 5B). Overall, electrophysiological variables were correlated with features of arbor size but not with branch level features such as bifurcation angles and tortuosity. In particular, the strongest marginal correlations were those between latency and a.totallength (ρ = 0.64), a.width and peak (ρ = 0.61), length and resistance (ρ = 0.56), a.width and amplitude (ρ = 0.58). While many electrophysiological variables strongly decreased with rel depth (e.g., ρ = −0.72 with peak), these variables were independent of rel depth conditional on up down ratio. As in the human BN, the Markov blanket of rel depth included a.height and up down ratio. Thus, as in human cells, the effect of cortical depth on the electrophysiology was not explained by depth-related differences in morphology. While resistance did decrease with apical and basal arbor size, the effect was somewhat weaker than in human cells (ρ = −0.48 with a.totallength).

Overall, the two BNs were different, with only four common arcs in their CPDAGs. No variable had an identical Markov blanket in the two Bayesian networks. The Hellinger distances were 0.91 and 0.85 on the human and mouse data sets, respectively.

### 3.4. Dependence on Cortical Depth

**Figure 6:**
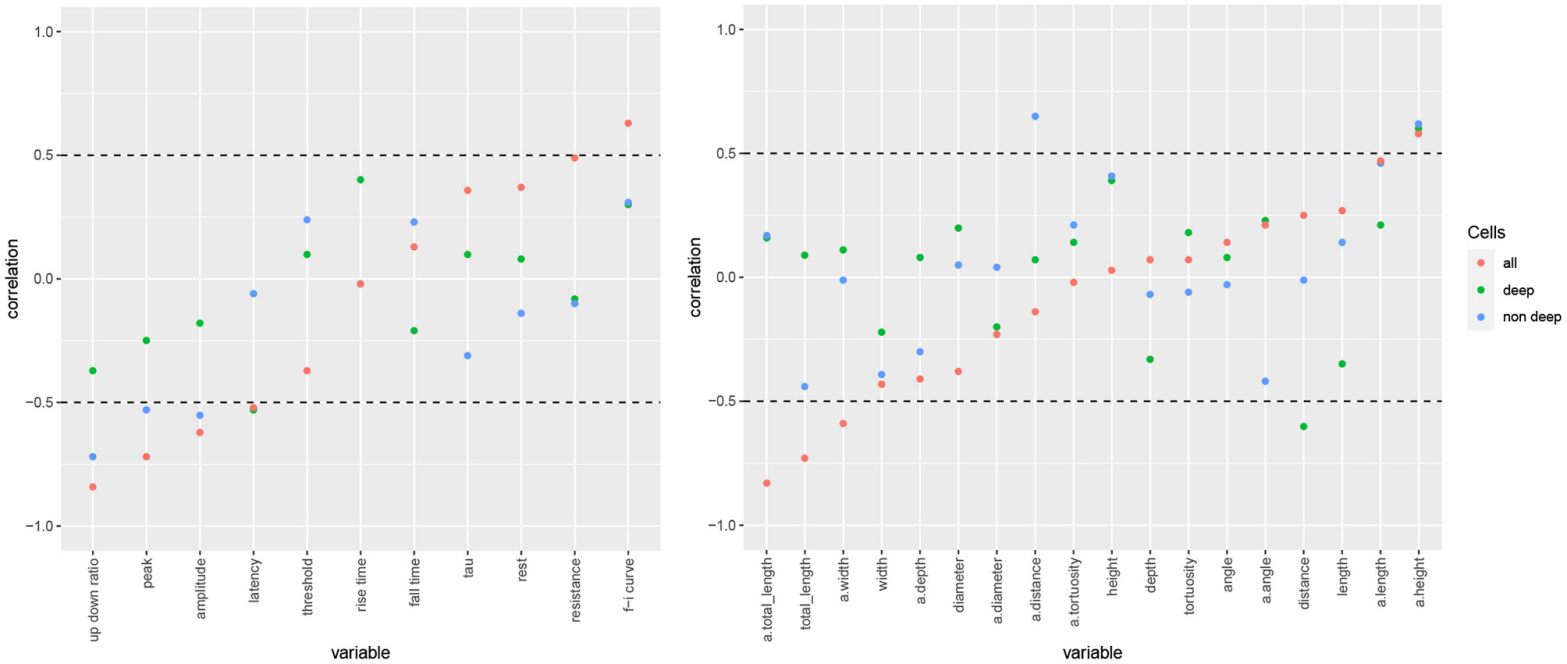
Electrophysiological **(left)** and morphological **(right)** variables' correlation with somatic cortical depth for all mouse cells (red), those located below a rel depth of 0.28 (deep) and those above it (superficial). Variables arranged by increasing overall correlation with rel depth, with horizontal lines at −0.5 and 0.5 separating the strong correlations that are shown in Figure 4.

We found that the negative correlation of rel depth and a.totallength in mouse neurons can be explained by the difference in length between cells located below a rel depth of 0.28 and those above it, as the deep cells had notably shorter apical arbors. In particular, a.totallength actually increased slightly with rel depth in both subgroups (ρ = 0.16 among deep cells and ρ = 0.17 among the non-deep cells, Figure 6) while the combined correlation was negative (ρ = −0.83). Likewise, rel depth was weakly correlated with a.width, diameter, resistance, threshold, and f-i curve within the subgroups yet strongly correlated overall (Figure 6). Thus, rather than varying smoothly with cortical depth, the observed dependences were fully or partially explained by the difference between deep and nondeep cells. On the contrary, rel depth was negatively correlated with latency in both subgroups yet not globally (Figure 6). For length as well as most action potential variables, the overall correlation was slightly stronger than among nondeep cells and notably stronger than among deep cells. For morphological variables unrelated to arbor size (e.g., angle and tortuosity), the correlation was rather consistent between the subgroups as well as globally. The correlation coefficients were largely similar between deep and nondeep cells, with a mean absolute difference of 0.26 and a maximum of 0.47 among electrophysiological variables (latency) and 0.65 among morphological variables (a.angle). Note that the subgroup estimates have high variance as there were 10 deep and 11 nondeep cells.

**Figure 7:**
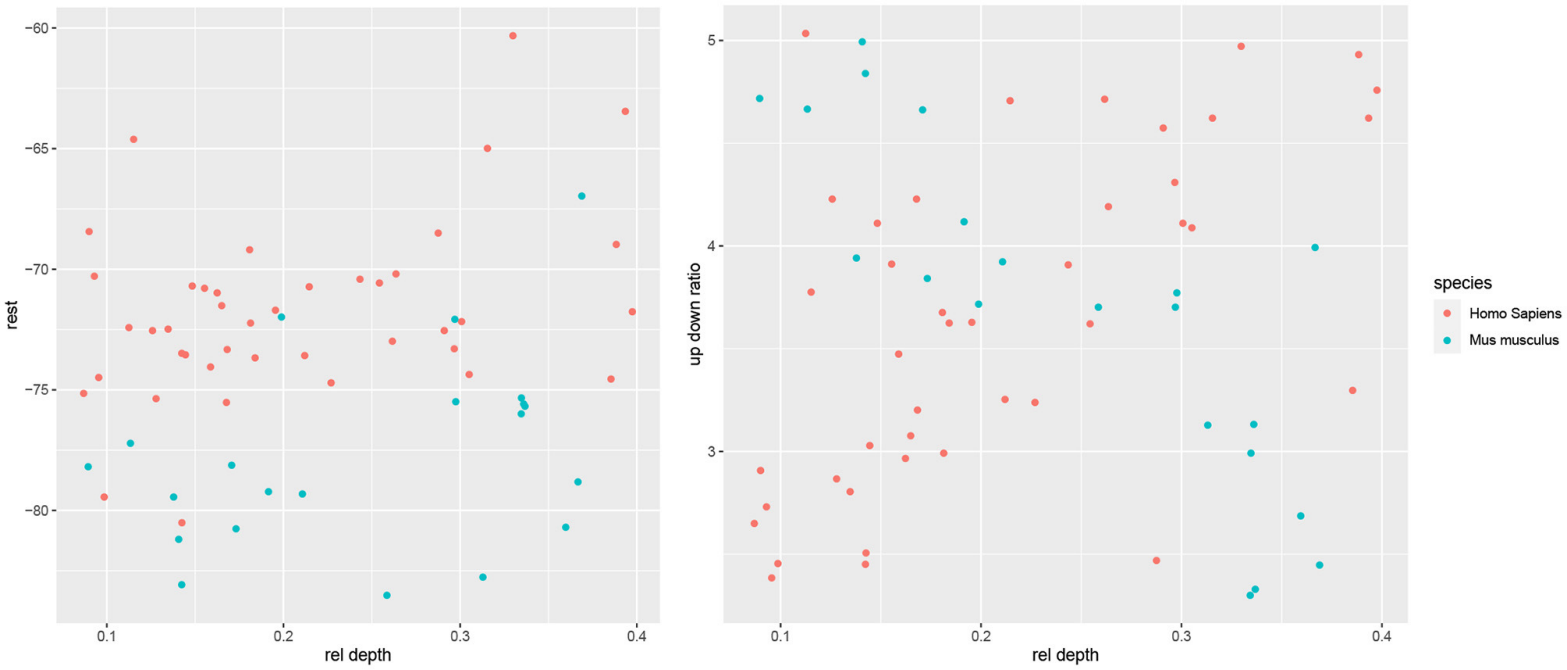
Scatter plots of rest **(left)** and up down ratio **(right)** with respect to rel depth.

Kalmbach et al. (2018) found that the cross-species differences in rest and resistance were depth dependent, with more difference among the most superficial L2 cells and the deepest L3 cells and less in the middle of L2/3. We did not formally test for such an effect, as there were too few mouse cells so as to bin them into groups according to cortical depth. However, visual inspection did not suggest such a dependence for the electrophysiological variables; instead, for most variables we observed a consistent difference across the L2/3 (one example is rest, Figure 7). An exception is up down ratio, which indeed differed only in the superficial and deep sections (Figure 7). In particular, up down ratio was higher among superficial mouse cells than among superficial human cells; it then decreased with rel depth for mouse cells yet increased for human ones, and thus did not differ between the two species toward the middle of L2/3 and was higher for human cells in the deep part of L2/3. Note that the means of up down ratio in the two species are similar and thus the t-test found no significant difference (Table 1).

## 4. Discussion

We found strong differences between the electrophysiology and morphology of human and mouse pyramidal neurons, both in terms of the variables' magnitudes and in terms of correlations between the variables, as evidenced by the differences in their Bayesian networks. In particular, the Hellinger distances ranged from 0.44 on electrophysiological variables to 0.91 on combined morphological and electrophysiological variables. While the maximal distance between two distributions is 1. We note that we compared Gaussian distributions with identical means.

We found strong correlations between electrophysiological and both apical and basal morphological variables in both species. In human cells, electrophysiological variables were not only correlated with morphological variables that are directly related to dendritic arbor size or diameter, but also to branch-level variables such as mean bifurcation angle and mean tortuosity. For some variables, we observed an opposite effect of cortical depth in the two species. We also found a strong effect of cortical depth on both morphology and electrophysiology in both species. In particular, the upstroke/downstroke ratio (up down ratio) increased with normalized cortical depth in human cells (ρ = 0.59) yet strongly decreased in mouse cells (ρ = −0.84). Likewise, while the length of the basal and apical arbors increased or stayed constant with cortical depth in human cells, it decreased strongly in mouse cells (ρ = −0.83 with a.totallength and ρ = −0.74 with totallength); notably, this was in spite of the apical height increasing with depth in mouse cells (ρ = 0.58). While Kalmbach et al. (2018) reported an effect of cortical depth on rest and resistance, we also report it for action potential properties such as up down ratio. We also showed that the correlation of electrophysiological features with cortical depth could not be explained in terms of the morphological variables. Overall, the effect of cortical depth differed between two species, perhaps reflecting differences in laminar organization of layers L2 and L3 between the two species. Our results suggest that, except regarding up down ratio, the cross-species differences are not depth dependent and that they hold across the depth of L2/3.

Our results regarding the effect of cortical depth are largely contrary to those by Deitcher et al. (2017), who found that electrophysiological features such as input resistance and membrane time constant were independent of depth in the human L2/3 pyramidal neurons of the temporal cortex (they did not assess the effect of cortical depth on electrophysiology in the mouse). Regarding morphology, they found that the size of the dendritic arbor increases with cortical depth in human pyramidal neurons but found no effect in mouse pyramidal neurons. Our results are, on the other hand, partially consistent with the results of Kalmbach et al. (2018). They found a positive correlation between rest and the rel depth in both species and a positive correlation between resistance and rel depth among mouse cells yet a negative one among human cells, albeit they could not confirm it in subsequent experiments, with a fixed membrane potential, for mouse cells. We confirmed the positive correlation with rest (ρ = 0.37 in both species), albeit weaker and only significant for the human cells, as well as the significant positive correlation with resistance in mouse cells (ρ = 0.49), yet only found a nonsignificant positive correlation in human cells (ρ = 0.13).

A possible explanation for our differences with the results by Deitcher et al. (2017) is that we had more electrophysiologically characterized human cells (42 vs. 25) and more morphologically characterized mouse cells (22 vs. 14), thus probably covering a wider range of somatic cortical depths and including the most superficial and deepest cells (Figure 1); indeed, this is the explanation proposed by Kalmbach et al. (2018) regarding a similar discrepance with Deitcher et al. (2017) in terms of cortical depth dependence of electrophysiology. Another difference in mouse cells is that we studied the visual cortex while (Deitcher et al., 2017) and Kalmbach et al. (2018) studied the temporal cortex. We note also that the patch clamp protocols were not identical in the three studies; however, it would not explain the differences with Deitcher et al. (2017) in the observed effect of cortical depth on morphology.

Our Bayesian networks are representative as long as the two samples are homogeneous, in the sense that the dependencies among variables are consistent across the cells of each sample. This may not be the case for mouse cells; for example, the correlation of latency and a.angle with rel depth varied between deep and nondeep L2/3 neurons, although that might be due to chance given the small sample sizes. Nonetheless, most deep cells indeed had distinctly smaller arbors and it is possible that at least some of them are star pyramidal neurons (Staiger et al., 2004); some of these L4 cells are also found in deep L2/3 in the Allen Cell Type Database. This depth-related difference in size could also be related to the distinction between profuse-tufted and slim-tufted neurons: Deitcher et al. (2017) noted that slim-tufted neurons tend to be located deeper in L2/3, although the separation was not as clear-cut as in our case. Nonetheless, when looking for two clusters with *k*-means and hierarchical clustering we obtained nothing similar to the distinction between deep and nondeep mouse cells.

Provided that our assumption of a multivariate Gaussian distribution of the variables holds, the learned Bayesian networks can be useful beyond identifying the independencies and correlations between variables. For example, they would allow for probabilistic reasoning regarding the morphology and electrophysiology of pyramidal neurons. For example, we could set the morphological variables to particular values and study the conditional distribution of electrophysiological variables. One might also use them for multioutput regression (Borchani et al., 2015), for example to predict the values of electrophysiological variables from those of the morphological variables.

## Data Availability Statement

Publicly available datasets were analyzed in this study. This data can be found at: Allen Cell Type Database.

## Author Contributions

BM designed and conducted the analysis and wrote the manuscript. All authors substantially reviewed the manuscript.

## Conflict of Interest

The authors declare that the research was conducted in the absence of any commercial or financial relationships that could be construed as a potential conflict of interest.
